# Institutional Notes and News

**Published:** 1911-01-28

**Authors:** 


### Institutional Notes and News.

**Worcester General Infirmary.**

There was a large attendance at the recent meeting of the Executive Committee, when the Bishop of Worcester was present. The attention of the Committee was called to the gratifying fact that many trade societies and kindred associations had increased their subscriptions this year. It was resolved to ask the Committee of the City of Worcester Memorial to King Edward VII. to appoint three or four of their number to form, with members of the Infirmary Committee, a Building Committee, who should appoint an architect and obtain estimates for the proposed memorial at the infirmary. Six members of the Committee were nominated on the part of the infirmary. A prolonged discussion took place on the question of certificates required by friendly societies and in cases arising under the Workmen's Compensation Act. The form of certificate in use at the Worcester Infirmary does not specify the nature of the disease from which the patient is suffering, and the representatives wish the form to be altered so as to supply that information. The medical staff object to such an alteration, on the grounds that such information could only be given with the patient's consent. Special certificates should be obtained at a charge to be arranged. The matter was referred to the House Committee for further consideration.

**Leicester firmary.**

A large attendance of governors and subscribers was present at the quarterly meeting of Leicester Infirmary to hear Sir Edward Wood move the adoption of the report last week. The work of the year has been continued at great pressure, the in-patients numbering 3,358 : in other words, an increase of 151 on the previous year. The average number of beds occupied has been 216, and the average stay per patient 26 days, while 43,803 out-patients have received treatment during the year and 46,509 attendances were recorded in the accident department; 519 severe accidents had been admitted from the latter department in the wards. The number of major operations on in-patients was 1,904, 1,111 operations had been performed under the hon. staff's supervision in the out-patients' department, and 1,668 anæsthetics had been administered in the accident department. This varied list of achievements is the cold summary in figures of the work done. The income for the year was £21,314, and we are glad to see that the annual subscriptions have increased slightly. The expenditure for the year amounted to £21,216, or £2,000 more than in the previous year, an excess due to the upkeep of the larger nurses' home and to larger purchases of bedding and linen and to the higher cost of uniforms. There have been many improvements, including the reconstruction of the *x*-ray department, the provision of new sewing rooms and extensive internal decorations. With the larger buildings spring cleaning has also proved more expensive. These facts are interesting because the board had made a careful estimate of the cost of maintenance of the institution for the year 1911, which they put at £22,500. In other words, an additional income of £1,500 was required, and if existing sources could be developed on lines which it specified, the board was certain that the institution could be maintained in a state of progressive efficiency. The Chairman, in moving the adoption of the report, alluded to the great necessity for a spare ward so that, among other contingencies, spring cleaning could be carried on without disorganising the work of the infirmary as was apt to be the case sometimes at present. Generally the year had been most successful, for notwithstanding the larger work, the income was sufficient to maintain the institution and to leave £100 in hand. Sir Edward Wood was thankful that annual subscriptions had developed from £3,073 seven years ago to £4,125, and he hoped eventually to see an income of £6,000 from this source. The increase in donations, most luckily, was owing to considerably more smaller sums, for which they were all most grateful. The great asset of the institution, however, Sir Edward went on, was the Saturday Hospital Fund, which this year had raised the magnificent sum of £13,600. The falling-off in legacies was time's revenge for the fact that 1909 had dealt bountifully with them. He looked forward to the time when all legacies could be capitalised. Efforts will be made during 1911 to institute systematic household servants' contributions.

**Southern Hospital, Manchester.**

The public of Manchester for some little time has been getting restive at the sight of a great disused building at the corner of Oxford Road and High Street. This is the empty Southern Hospital, better known, perhaps, because of its amalgamation with other institutions, as the St. Mary's Hospitals for Women and Children. It is not long since the new building has been completed; every equipment, apparently, has been provided, but the hospital remains tenantless to-day. A fitful correspondence in the Manchester papers has now drawn Mr. Harold Agnew, the treasurer, to give the true explanation of the present state of affairs. It appears that £13,000 was received from an appeal, and an increase of £250 in annual subscriptions, but "the Board has satisfied itself that an increased income of from £4,000 to £5,000 is necessary to carry on the work satisfactorily." Most fortunately, during the past few months the Board has received several handsome promises of donations, and as early as this April the new branch that is opposite Whitworth Park, is to be opened. It has been felt that no blame attaches to the board of management for the delay in opening the hospital, since the board built the hospital and equipped it, but, unless we are mistaken, the money for these purposes was provided largely by a single donor. Once equipped, however, "with a scrupulousness," as the *Manchester Guardian* slyly remarks, "not always associated with institutions of charity, they (the board) have abstained from opening it till they can see the possibility of paying for its maintenance." That, no doubt, is the reasonable view, but it does not require a very acute observer of recent events to see that the main impetus towards the successful raising of the money needed for this maintenance has come from a vigorous letter by the Rev. J. E. Roberts, who says "very often the surest way to secure support is to challenge the constituency by a bold action. The sight of neglected buildings depresses. If Manchester people could see the wards filled with needy patients, would not many of them come to the help of the committee?" This is the unreasonable view, which the interest its statement has aroused already shows also to be the successful one. We think that Mr. Roberts has done the St. Mary's Hospitals a very good turn, and we hope that the board of management will take the hint they have received, and make the meeting which the Lord Mayor has promised to hold in the Town Hall on February 2, the beginning of a great financial success.

